# Mortality in Catalonia during the summer of 2022 and its relation with high temperatures and COVID-19 cases

**DOI:** 10.3389/fpubh.2023.1157363

**Published:** 2023-05-19

**Authors:** Ermengol Coma, David Pino, Núria Mora, Francesc Fina, Aida Perramon, Clara Prats, Manuel Medina, Antoni Planella, Anna Mompart, Jacobo Mendioroz, Carmen Cabezas

**Affiliations:** ^1^Primary Care Services Information System (SISAP), Institut Català de la Salut (ICS), Barcelona, Spain; ^2^Department of Physics, Universitat Politècnica de Catalunya, Barcelona, Spain; ^3^Department of Health, Generalitat de Catalunya, Direcció General de Planificació i Recerca en Salut, Barcelona, Spain; ^4^Department of Health, Public Health Secretariat, Generalitat de Catalunya, Barcelona, Spain

**Keywords:** mortality, COVID-19, influenza-like illness-ILI, heat, hot temperature

## Abstract

**Purpose:**

To analyse the association between the mortality during the summer 2022 and either high temperatures or the COVID-19 wave with data from the Catalan Health Care System (7.8 million people).

**Methods:**

We performed a retrospective study using publicly available data of meteorological variables, influenza-like illness (ILI) cases (including COVID-19) and deaths. The study comprises the summer months of the years 2021 and 2022. To compare the curves of mortality, ILI and temperature we calculated the *z*-score of each series. We assessed the observed lag between curves using the cross-correlation function. Finally, we calculated the correlation between the *z*-scores using the Pearson correlation coefficient (*R*^2^).

**Results:**

During the study period, 33,967 deaths were reported in Catalonia (16,416 in the summer of 2021 and 17,551 in the summer of 2022). In 2022, the observed lag and the correlation between the *z*-scores of temperature and all-cause deaths was 3 days and *R*^2^ = 0.86, while between ILI and all-cause deaths was 22 days and *R*^2^ = 0.21. This high correlation between temperature and deaths increased up to 0.91 when we excluded those deaths reported as COVID-19 deaths, while the correlation between ILI and non-COVID-19 deaths decreased to −0.19. No correlation was observed between non-COVID deaths and temperature or ILI cases in 2021.

**Conclusion:**

Our study suggests that the main cause of the increase in deaths during summer 2022 in Catalonia was the high temperatures and its duration. The contribution of the COVID-19 seems to be limited.

## Introduction

During summer 2022, according to EuroMOMO mortality monitoring system data, Spain presented an extraordinarily high excess mortality (*z*-score up to 14.1). This occurred especially in July and among the population aged over 75 years. Although Spain was one of the countries with the highest excess mortality during that summer, other countries such as France, Germany, Italy, and England also experienced an excess mortality (*z*-score between 7 and 10) (1).

The daily “mortality monitoring system for all causes” (MoMo), published since 2004 by the Instituto Carlos III (Spain), estimates the expected deaths and compares them to those observed in Spain. The estimation of expected deaths is calculated daily using data from the last ten years (excluding 2020, because of the pandemic exceptionality) (2). The MoMo report of September 14, 2022 (3) showed 44,754 deaths in July throughout Spain, which represents an excess of 11,349 deaths (34% of excess compared to the expected). Conversely, in June and August, the excess mortality was lower, with an excess of 4,454 (13.6% excess) and 5,293 deaths (16%), respectively (3). Overall, the excess for the three months was 21.096 deaths (21%).

Catalonia, a region in the north-east of Spain with a population of 7.76 million people, also presented an excess mortality during the 2022 summer months (June–August) of around 17% (12.4% in June, 25.5% in July and 12.8% in August), that was one of the lowest excess deaths in Spain and four points below the Spanish average (3). Conversely, other regions such as Navarra or Extremadura presented more than 30% excess deaths during the summer months. The excess in Catalonia was mainly in July and concentrated in the population over 75 years (4).

The excess mortality was also observed in other European countries, but there are still uncertainties about the leading causes. For instance, the Office for National Statistics analysed the excess in England and Wales (5) and they observed an increase in deaths of 10.2% above the July five-year average (2016 to 2019, and 2021). Mortality during July 2022 in England was attributed to dementia, followed by ischemic heart disease, malignant neoplasm and cerebrovascular disease. Coronavirus disease (COVID-19) was the sixth leading cause of death in July 2022 in both England (accounting for 3.8% of all deaths) and Wales (4.0% of all deaths), significantly increasing the standardised mortality rate for deaths due to COVID-19 between June and July 2022.

Heat waves affected different European countries during summer 2022. June, July, and August 2022 were the third, sixth and first warmest June, July, and August on record in Europe, respectively (6). The heat waves especially affected France, Italy and Spain in June, but extended to the United Kingdom (UK), Central Europe and Scandinavia in July and August. Additionally, these heat waves lasted longer than previous heat waves, particularly in southwestern Europe, where July 2022 was the warmest July on record for the average maximum temperatures and the third warmest July according to minimum average temperatures (7).

In Spain, summer 2022 was the warmest summer since 1961, with a mean temperature anomaly of +2.2°C with respect to the 1971–2000 period, 0.4°C above the second warmest summer in this period, 2003. According to the Spanish Meteorological Agency (AEMET), three heat waves^1^ were recorded during the summer: 12–18 June, 9–26 July, and 30 July-15 August. The second heat wave was the second longest and extended heat wave of the recorded series, affecting 48 provinces. It was the most intense (largest temperature anomaly) of all the recorded heat waves. The third one (30 July–15 August) was the third longest heat wave of the series (8).

These three heat waves affected the four provinces of Catalonia (9). According to the data of the Catalan Meteorological Service, in 107 of the automatic weather stations, summer 2022 was the warmest summer in the last 20 years. For the rest of the stations it was the second warmest summer. Considering the data of two centennial meteorological stations (Ebre and Fabra Observatories), summer 2022 was the warmest summer in the last 105 years and the second warmest since 1915, respectively (10, 11).

Positive temperature anomalies, and in particular heat waves, are one of the major causes of excess mortality (12, 13), especially in older people (14–16). Not only because acute heat stress increases mortality by itself, but also because it especially increases the mortality associated with cardiovascular, respiratory or cerebrovascular previous conditions (14, 17–19). This was clearly observed in Europe during the heat wave that occurred in the summer of 2003 (20–24). During summer 2022 (25), modelled the association between MoMo daily mortality and daily mean temperature for the 52 Spanish capital cities. The analysis attributes 706, 3,204, and 1,406 deaths to extreme heat and 1798, 2,588 and 2,352 deaths to moderate heat in June, July, and August 2022, respectively. Additionally, climate change projections indicate that heat wave frequency will increase in the coming decades and their impact will be higher in the southernmost Europe (26).

However, since the beginning of the COVID-19 pandemic, excess mortality has been generally associated with increased transmission of SARS-CoV-2, and, as in the case of high temperatures, mortality has been centred mainly on the older adults (27–29). Additionally, the pandemic appears to have increased the heat-related mortality (30), despite SARS-CoV-2 transmission appears to be reduced at high temperatures (31).

The exact cause of the deaths in Spain will not be available until the end of 2023, as it is considered statistical information and not health data. During the summer 2022, Catalonia also experienced both several prolonged heat waves and a COVID-19 wave [mainly due to Omicron BA.5 according to official data (32)]. In our study, we used publicly available data to analyse the possible association between the increased mortality in Catalonia during the summer months of 2022 and either higher than average temperatures or the COVID-19 wave. In addition, we performed the same analysis for summer 2021 in order to compare both pandemic years.

## Materials and methods

### Study design and data sources

We conducted a population-based retrospective study. The meteorological data was obtained from the network of automatic weather stations (AWS) of the Servei Meteorològic de Catalunya (Catalan Meteorological Service). This network includes 186 weather stations that cover all the Catalan territory. We obtained from the Catalan Open Data website^2^ the hourly temperature (°C) and relative humidity (%) recorded at the stations since 2012.

Data on daily influenza-like illness (ILI) cases, COVID-19 cases and COVID-19 deaths were obtained from the official Catalan infections surveillance system (SIVIC for its Catalan initials: Sistema d’informació per a la vigilància d’infeccions a Catalunya) (32), available at https://sivic.salut.gencat.cat/dades_obertes. Finally, daily all-cause mortality was obtained from the official register of insured persons (RCA for its Catalan initials: Registre Central d’Assegurats) that includes all of the Catalan population, publicly available in the Catalan Open Data website.^3^

### Population and study period

Our study includes the entire Catalan population. The analysed time period comprises the meteorological summer months of the years 2021 and 2022, from 1^st^ June to 31^st^ August. Meteorological data from previous years (2012 to 2020) was used to analyse the lag between high temperatures and mortality. In addition, the number of deaths and ILI cases since 2012 were also obtained as context.

### Outcomes and variables

The main outcome was mortality assessed by the number of daily deaths during the study period. Crude mortality rates (CMR) per 100,000 people were calculated by dividing the number of deaths in each summer by the population size. The population size was extracted from the RCA and it included the number of alive people the day before each summer period. To calculate the standardised mortality rate (SMR) we performed a direct standardisation method by sex and age groups.

The daily averaged temperature and relative humidity for each AWS were calculated averaging the hourly data. Subsequently, we excluded the records of those AWS located >1,500 metres above sea level [*N* included = 166 (89%)].

Considering the impact of high temperatures (15, 16, 33) and COVID-19 (27, 29) in different cohorts, the analysis was performed globally, by different age groups (0–44 years old, 45–79 years old and > 79 years old) and different health regions classified according to whether they include coastal areas or not. As for the latter, the meteorological variables were averaged for each region.

### Statistical analyses

All series were computed as a 7 day moving average to avoid weekly effects on recording practice that otherwise affect the frequency of ILI and COVID-19 cases (34).

To compare the curves of mortality, ILI and temperature, we calculated the *z*-score of each series during the study period. To assess the observed lag between curves, we used the cross-correlation function which allows for identifying the most correlated time lag between two time series.

The correlation between the *z*-scores of ILI or temperature and mortality was calculated using the Pearson correlation coefficient once the theoretical lags have been applied. The identification of theoretical lags is explained in the Supplementary Material S1.

Although all analyses have been performed using temperature, we have also analysed the role of humidity in the excess mortality of summer 2022 by calculating, using temperature and relative humidity, an apparent temperature or heat index (35) for each hour and weather station. During summer 2022 in Catalonia, humidity did not play a key role in explaining excess mortality (see Supplement Material S2).

All analyses were performed using R version 3.5.1 (36).

## Results

During the study period, 33,967 deaths were reported in Catalonia, 16,416 in the summer of 2021 and 17,551 in the summer of 2022, which represented an SMR of 198.4 and 207.0 deaths per 100,000 people, respectively. Although the number of deaths increased by 6.9% in the summer of 2022 compared to 2021, the standardised rates were only 4% larger, similar to those of 2013, 2014 and 2016, and smaller than those of 2012 and 2015. The percentage of COVID-19 deaths was around 7.8% during the summers of 2021 and 2022, and the percentage of ILI that were COVID-19 confirmed cases was smaller during the summer of 2022 than during the previous two years (51% vs. 67%). Table 1 summarised this information since 2012.

**Table 1:** Population, number of deaths, crude and standardised mortality rates, number of COVID-19 deaths, number of influenza-like illness (ILI) cases and number of COVID-19 cases during the summer months (June to August) by year in Catalonia.

Year	Population	Number of deaths (All causes)	CMR (per 100,000)	SMR (per 100,000)	Number of COVID-19 deaths	Number of ILI cases	Number of COVID-19 cases
2012	7,570,579	14,770	195.1	211.1	0	136,613	0
2013	7,540,378	15,018	199.2	210.0	0	182,463	0
2014	7,527,714	15,133	201.0	206.2	0	208,315	0
2015	7,472,216	15,972	213.8	213.8	0	203,468	0
2016	7,454,318	15,558	208.7	203.1	0	223,274	0
2017	7,458,944	14,782	198.2	188.3	0	231,047	0
2018	7,488,690	15,467	206.5	193.2	0	228,612	0
2019	7,541,662	15,066	199.8	185.6	0	220,567	0
2020	7,613,755	15,172	199.3	188.3	680	237,141	158,781
2021	7,667,071	16,416	214.1	198.4	1,290	481,078	320,675
2022	7,764,830	17,551	226.0	207.0	1,366	495,928	251,244

CMR: crude mortality rate; SMR: standardised mortality rate.

Regarding age and sex, we observed that during the summer of 2022 the percentage of deaths with more than 79 years of age was higher than those of pre-pandemic years (64.1% in 2022 vs. an average of 61.2% in the 2012–2019 period), while the percentage of deaths in women was also slightly higher in 2022 (51.1% vs. 50% in the 2012–2019 period) (Supplementary Table S1).

**Figure 1:**
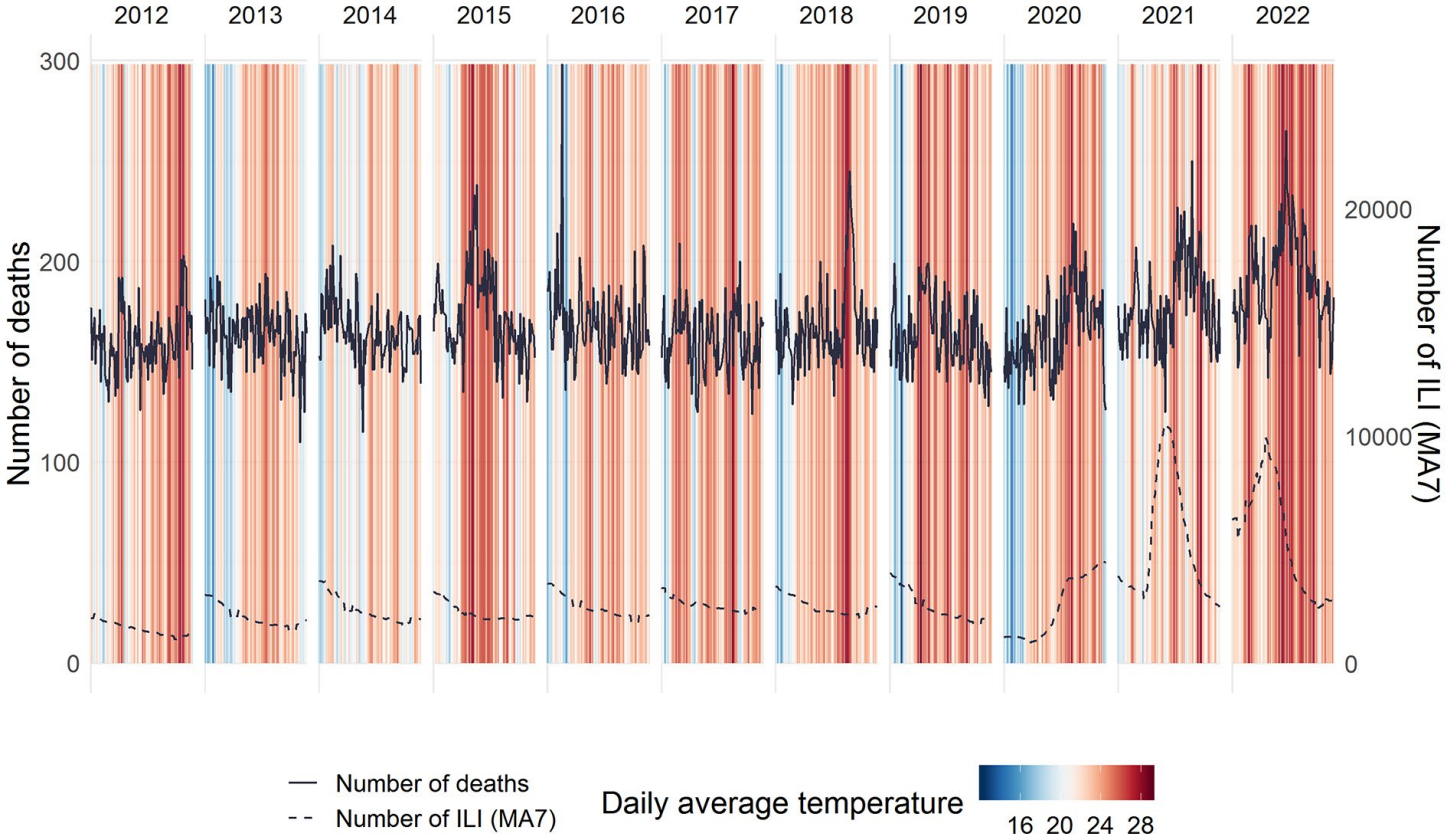
Daily warming stripes, number of deaths (solid lines) and number of influenza-like illness (ILI, dashed lines) cases during the summer months (June–August) by year.

Figure 1 shows the evolution of the daily average temperature (colour stripes) during the summer months since 2012. We observed that the 2022 summer had more days with daily mean temperature in the highest percentile of the series (red stripes). During the summer of 2015 high temperatures were also recorded, although for fewer days than in 2022. In fact, the number of days with the highest temperature above 33°C was 29 in 2022 versus 14 in 2019, the second year with the most days of high temperatures. In 2021, this number of days was 9. These high temperatures in 2015, 2018 and, especially, 2022 coincided with an increase in the number of deaths (solid black line) during those summers. In addition, before the COVID-19 pandemic, the number of ILI cases (dashed black line) during summer months was at baseline levels.

**Figure 2:**
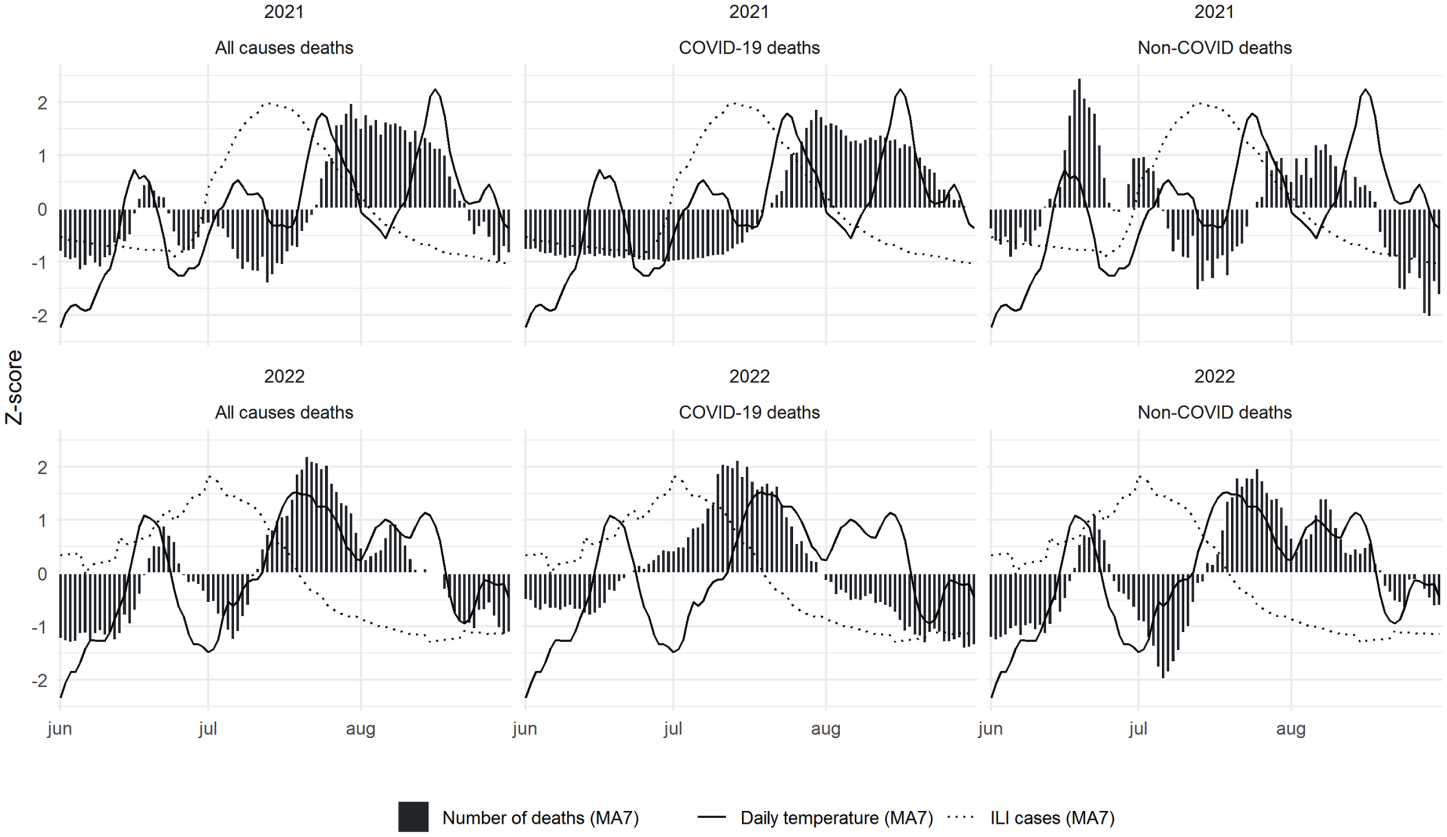
*Z*-scores of deaths (bars), temperature (solid line) and ILI cases (dashed line) by cause of death (all causes, COVID-19 and non-COVID deaths) and year. Data is presented using a 7 day moving average.

To compare the number of deaths, ILI cases and temperature we calculated the *z*-scores for each series (Figure 2). We observe that, if a small lag is considered, the days with the highest overall number of all-cause deaths in the summer of 2022 coincided with the days with the highest temperatures. On the contrary, the days presenting the largest number of ILI cases occurred earlier. Moreover, the main increase in COVID-19 deaths in 2022 occurred before the increase in all-causes deaths and represented a small fraction of the total summer deaths (Table 1). Finally, as shown in Figure 2, in 2021 the relation between the three variables is less evident. This pattern is not observed for the population under 45 years old (Supplementary Figure S1).

Analysing the different *z*-score curves, we estimated that the lags between temperature and all-cause deaths and between temperature and non-COVID deaths were both 3 days in 2022, coinciding with the lag observed in previous works (37–39)(see Supplementary Material S1). However, these lags in 2021 were 0 and −1 days, respectively, significantly lower than the reference used in our analysis. On the other hand, the lags between ILI cases and all-cause deaths and between ILI cases and non-COVID deaths were 22 and 28 days, respectively, in 2022; and 21 and 22 days in 2021, far away from the reference lags calculated (see Supplementary Material S1).

**Figure 3:**
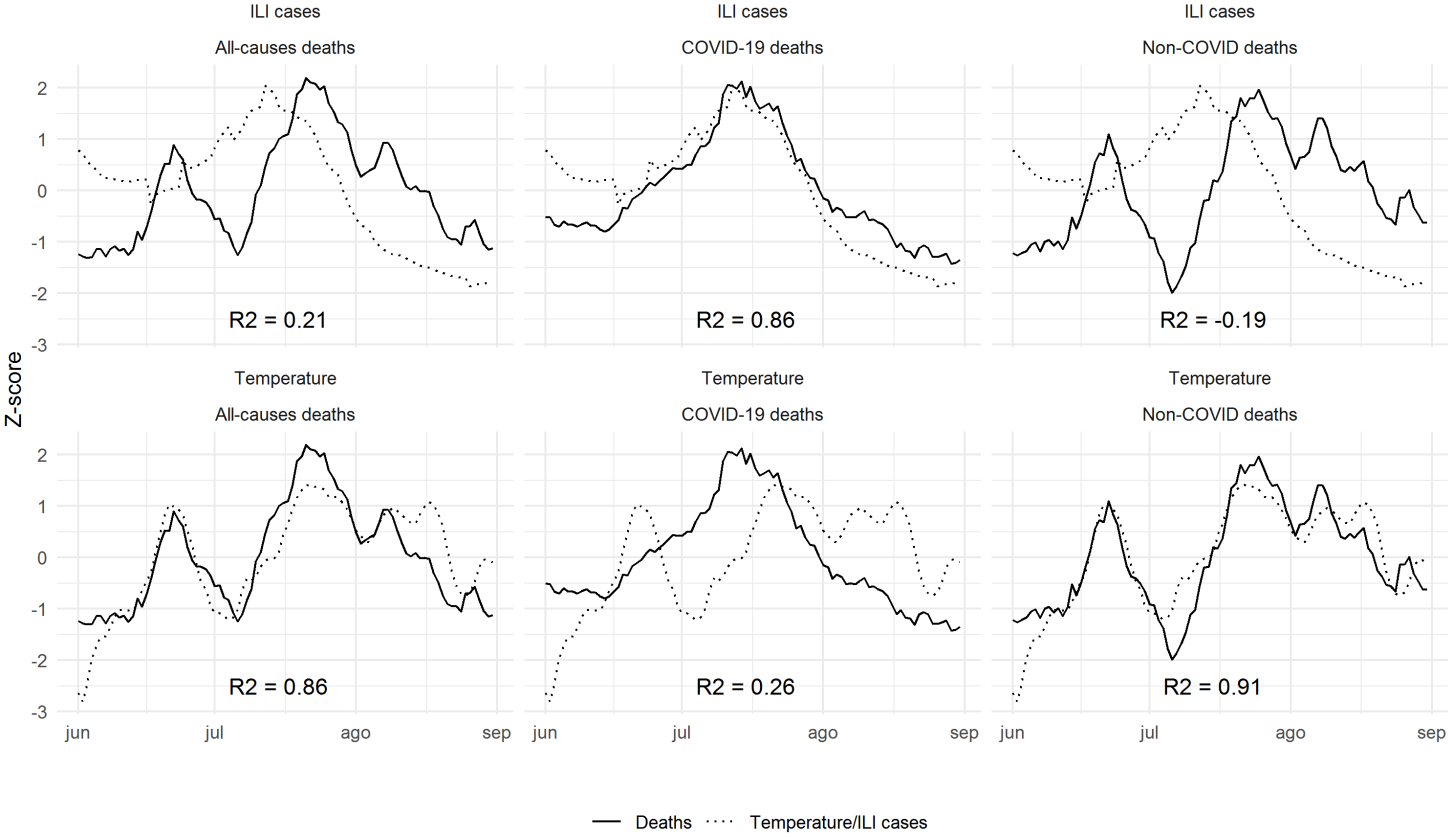
Correlations between *z*-scores of deaths (solid lines) and temperature/ILI cases (dashed lines) by cause of death (all-causes, COVID-19 deaths and non-COVID deaths) during summer 2022, once the theoretical lags have been applied.

Once the lags had been applied to the *z*-score curves (3 days between temperature and deaths and 11 days between ILI and deaths, as stated in Supplementary Material S1), the correlation between temperature and all-cause deaths in summer 2022 was 0.86, while the correlation between ILI and all-cause deaths was 0.21. This high correlation in 2022 between temperature and deaths increased up to 0.91 when we excluded those deaths reported as COVID-19 deaths, while the correlation between ILI and non-COVID-19 deaths decreased to −0.19. Finally, we observed a high correlation between ILI cases and COVID-19 deaths (0.86) and a low correlation between temperature and COVID-19 deaths (0.26) (Figure 3). These findings were similar when we replicated the analysis using a heat index that includes temperature and relative humidity. The correlation between apparent temperature (heat index) and all-causes deaths was 0.86 and between apparent temperature and non-COVID deaths was 0.92, very similar to the main analysis (Supplementary Figure S2).

This was consistent for the two oldest age groups (45–79 years and > 79 years). Nonetheless, we found no significant correlation between temperature or ILI cases and deaths in the population below 44 years old (Supplementary Table S2). These results were different in 2021, where all-cause deaths presented a moderate correlation for both ILI cases and temperature (0.43 with ILI and 0.49 with temperature). No correlation was observed between non-COVID deaths and temperature or ILI cases in 2021 (Supplementary Table S2).

Finally, the correlation between temperature and non-COVID deaths was higher in health regions located in coastal areas than in those located in non-coastal areas (0.86 vs. 0.61), while correlation with ILI cases was low in both areas (Supplementary Table S3).

## Discussion

Our analysis of the *z*-scores curves of temperature/ILI and deaths points to a high correlation between the high temperatures recorded during summer 2022 and all-cause deaths in Catalonia, Spain. Correlation increased when deaths reported as COVID-19 deaths were excluded from the analysis, suggesting both that the prolonged heat wave experienced in Spain during this summer was the most plausible explanation for the excess of deaths observed, and that there was not an infra-detection of COVID-19 deaths. The absence of correlation between ILI cases and non-COVID deaths and the high correlation between ILI cases and COVID-19 deaths points in the same direction. In addition, data from summer 2021, when there were only two heat waves with lower temperature anomaly and duration (40), and no high correlation between temperature and deaths was found, reinforces these conclusions. Furthermore, the delay observed in summer 2022 between the curves of temperatures and deaths is consistent with previous studies (41), while the lag between ILI and deaths is more than double that described in other works (42).

The association between extremely high temperatures and mortality has been already analysed for previous heat waves in Europe (21, 43–47), but, obviously, without experiencing a pandemic during the same period. Focusing on Spain, in summer 2003 there occurred two heat waves: 20–23 June and 30 July-14 August. The first one had a small duration, extension and temperature anomaly, but the second one presented similar characteristics to two of the three heat waves in 2022 (see Supplementary Table S4). That summer mean temperature in Spain was 23.6°C, the second highest mean summer temperature of the Spanish series, just below the summer 2022, whose mean temperature was 24°C. The excess of mortality during summer 2003 only occurred for people above 75 years (48). The percentage of excess in Spain was 15% for the cohort 75–84 and 29% for people over 85 years. This excess during summer months for these age groups was higher in the summer of 2022 (Supplementary Table S5) (3).

The MoMo report also estimates the percentage of excess deaths attributable to high temperatures: in July 2022, only 5.0% (2,223) of the total deaths in Spain were attributable to high temperatures (3). Nonetheless, the MoMo uses a predictive model based on the Kairós index that takes into account the maximum and minimum daily temperatures by associating a relative risk with three levels of excess mortality: small or zero, moderate and high (2). This model may not adapt to changes in the behaviour of heat waves in 2022 compared to previous years, with an earlier onset and longer, more numerous and persistent waves, and of different intensity and meteorological characteristics (40). Nor is the population’s response to these waves the same, in a new context characterised by the increase in energy poverty. These behavioural changes could lead to associating a lower relative risk and, consequently, underestimate the 2022 deaths attributable to current heat waves.

Since the beginning of the COVID-19 pandemic, the number of ILI cases during summer months has doubled, especially during 2021 and 2022. In consequence, this could have some effect on mortality during the two last summers. Although it is not clear yet how the dynamics of SARS-CoV-2 will be from now, future estimations of the excess mortality should consider this increase in ILI cases during the summer months that did not happen before the pandemic. Nonetheless, when we compare standardised mortality rates since 2012, we observe that 2022 is the fourth year with more mortality during summer and 2021 is the seventh. We have to take into account that mortality (and excess deaths) should be analysed in large periods in order to interpret it. For instance, according to MoMo data (3), during the first 3 months of 2022 in Catalonia, observed deaths were only 1.5% higher than expected mainly due to the late and weaker influenza epidemic and to the fact that the COVID-19 wave was driven by Omicron variant, less severe than Delta variant (49, 50). This could result in a more susceptible population who was thereafter hit by a prolonged heat wave during summer months.

In our study we focused on two well-known causes of excess mortality previously described in the literature, such as high temperatures and respiratory infection epidemics (48, 51, 52). However, other causes may play a role and consequently ongoing research about the topic is needed. In addition, socioeconomic losses and the increase in inequalities during the COVID-19 pandemic could have increased vulnerability of some populations and exacerbated the impact of a heat wave (27). More analysis should be performed to further understand this point. Finally, other elements such as disruptions in healthcare and the delay in screenings and diagnoses [for instance, in Catalonia we observed a large reduction in cancer diagnoses during the COVID-19 pandemic (53, 54)] should also be considered and should be studied in depth in future research. All these factors could have contributed to an increase in overall mortality, but they are unlikely to be the main cause of the excess mortality observed during the summer of 2022.

While our ecological analysis has the known limitation of making individual inference impossible, it is useful for raising hypotheses about the most plausible cause of summer-2022’s excess deaths. As long as the cause of each death is not available yet, this is a valid approach to the topic that could be reproduced in other regions. In order to confirm our findings, further studies should be performed once the Spanish National Institute of Statistics publishes the cause of deaths. Another limitation of the study is related to the use of temperature as the only meteorological variable to relate to mortality, without including any percentile of temperature or different thresholds to define a heat wave (22, 55). Additionally, previous studies have used atmospheric variables different from temperature that seem to provide better correlation with excess mortality. Some of them, following (35), used a combination of temperature and a variable related to humidity to take into account how the effectiveness of sweating to decrease body temperature is impaired in high relative humidity conditions (41, 56) or used different temperature metrics (55, 57). In our opinion, humidity played a less important role than temperature in explaining excess mortality during summer 2022 in Catalonia. Moreover, its role may change depending on the regional climate or on the specific temperature anomaly of the heat wave. In our study, we also used a heat index to correlate with excess mortality without observing a better correlation. In addition, mean temperature has been also used to analyse mortality during heat waves because it can be easily interpreted by policy makers (37, 58). Finally, COVID-19 testing protocols were different during summer 2022 and summer 2021. For this reason, in our main analysis we used ILI cases to reduce the possible effect of an underdetection of COVID-19 confirmed cases.

Beyond these limitations, this study also has strengths. We used multiple datasets with extended (hourly temperature, daily cases and deaths) and complete data from a large period that allowed us to perform an accurate analysis. In addition, these existing and publicly available databases cover the entire Catalan region and the analyses could be performed in other regions with the available information.

In conclusion, our study suggests that the main cause of the increase in deaths during summer 2022 in Catalonia was the high and prolonged temperatures during the period. The contribution of the COVID-19 pandemic to the excess mortality, based on the data analysed, seems to have been limited. In addition, our analysis also suggests that there is no infra-detection of COVID-19-related deaths in the official figures.

## Data availability statement

Publicly available datasets were analyzed in this study. This data can be found here: https://analisi.transparenciacatalunya.cat/Medi-Ambient/Dades-meteorol-giques-de-la-XEMA/nzvn-apee, https://sivic.salut.gencat.cat/dades_obertes and https://analisi.transparenciacatalunya.cat/Salut/Defuncions-a-Catalunya/3cre-fa8v.

## Ethics statement

Ethical review and approval was not required for the study on human participants in accordance with the local legislation and institutional requirements. Written informed consent for participation was not required for this study in accordance with the national legislation and the institutional requirements.

## Author contributions

EC, DP, NM, FF, MM, AM, JM, and CC: conceptualisation. EC, DP, NM, FF, APe, and CP: methodology. EC, NM, and FF: validation. EC, NM, DP, and FF: formal analysis. EC, DP, NM, FF, APe, and CP: writing—original draft preparation. EC, DP, NM, FF, APe, CP, MM, APl, AM, JM, and JM: Interpretation and visualisation. EC, DP, MM, AM, JM and CC: supervision. EC, FF: guarantors. All authors contributed to the article and approved the submitted version.

## Funding

APe received funding from Fundació La Marató de TV3 through the grant 202134-31.

## Conflict of interest

The authors declare that the research was conducted in the absence of any commercial or financial relationships that could be construed as a potential conflict of interest.

## Publisher’s note

All claims expressed in this article are solely those of the authors and do not necessarily represent those of their affiliated organizations, or those of the publisher, the editors and the reviewers. Any product that may be evaluated in this article, or claim that may be made by its manufacturer, is not guaranteed or endorsed by the publisher.

## Supplementary material

The Supplementary material for this article can be found online at: https://www.frontiersin.org/articles/10.3389/fpubh.2023.1157363/full#supplementary-material

## Footnotes

^1^AEMET defined a ‘Heat wave’ as an episode of at least three consecutive days, in which at least 10% of the stations considered record maximums above the 95th percentile of their daily maximum temperature series for the months of June, July and August of the period 1971–2000.

^2^ https://analisi.transparenciacatalunya.cat/Medi-Ambient/Dades-meteorol-giques-de-la-XEMA/nzvn-apee

^3^ https://analisi.transparenciacatalunya.cat/Salut/Defuncions-a-Catalunya/3cre-fa8v
