# Supplementary material for: Mortality in Catalonia during the summer of 2022 and its relation with high temperatures and COVID-19 cases
Source: Front Public Health. 2023 May 19;11:1157363. doi: 10.3389/fpubh.2023.1157363 (PMC10235629; doi:10.3389/fpubh.2023.1157363)
Supplement: Supplementary file 1 [file Data_Sheet_1.pdf]

Supplementary material

Mortality in Catalonia during the summer of 2022 and its relation with high temperatures and COVID-19 cases

Ermengol Coma, David Pino, Núria Mora, Francesc Fina, Aida Perramon, Clara Prats, Manuel Medina, Antoni Planella, Anna Mompart, Jacobo Mendioroz, Carmen Cabezas

Table of contents

Supplementary material S1. Calculation of the theoretical lags between COVID-19 cases and mortality and between temperature and mortality. 2

Supplementary material S2. Formulae used to calculate apparent temperature 3

Supplementary Table S1. Number of deaths by sex and age groups during the summer months since 2012 4

Supplementary Figure S1. Z-scores of non-COVID deaths, COVID-19 deaths, ILI cases and temperature by age group and year. MA7 = 7-day moving average 5

Supplementary Figure S2. Correlations between z-scores of deaths (solid lines) and apparent temperature/ILI cases (dashed lines) by cause of death (all-causes, COVID-19 deaths and non-COVID deaths) during summer 2022, once the theoretical lags have been applied 6

Supplementary Table S2. R2 between the z-scores of ILI cases and deaths and between temperature and deaths (in bold those correlations above 0.8) 7

Supplementary Table S3. R2 between the z-scores of ILI cases and deaths and between temperature and deaths by regions (in bold those correlations above 0.8) 9

Supplementary Table S4. Characteristics of the heat waves of 2003 and 2022 (source AEMET) 10

Supplementary Table S5. Characteristics of the excess deaths* of 2003 [Simón et al. 2005] and 2022 [MoMo, 2022] between 1 June and 31 August in Spain 11

Supplementary material S1. Calculation of the theoretical lags between COVID-19 cases and mortality and between temperature and mortality.

Taking into account there is a lag between the curves of ILI/COVID-19 cases and deaths and temperature and deaths, it is important to calculate this theoretical lag before performing the correlation between the series.

To assess the theoretical lag between COVID-19 cases and deaths we calculated the lags between COVID-19 cases and COVID-19 deaths during 2022, when the Omicron variant was predominant, using the cross-correlation function. The lag is estimated as the point where the correlation between curves is maximum. The lag observed between COVID-19 cases and COVID-19 deaths during 2022 was 11 days. This is consistent with the lag of 10 days between COVID-19 confirmed cases and deaths observed in a previous study performed in 2020[1] [Català et al., 2021]. In addition, another study[2] analysing the time between diagnosis of COVID-19 and death (Diagnosis to Death, DtD) during the first wave of COVID-19, found that the maximum of correlation was country dependent, although all observed times were < 11 days. In fact, for countries that have not seen a clear correlation as Sweden authors used a range of possible intervals between 4 and 14 days. Both references support the use of the theoretical lag considered in this manuscript.

Regarding the mortality associated with high temperatures, its effects occur in 1-2 days[3],[4] [Nastos and Matsarakis, 2011; Wang et al., 2014] or from 0 to 5 days from the day of exposure[5] [Luo et al., 2019]. To calculate the theoretical lag between temperature and deaths, we calculated the lag during the summer periods since 2012. This lag was 3 days in those years with heat waves (2015, 2017, 2018) and it is consistent with previous literature[6] [Royé et al., 2019].

---

[1] Catala M, Coma E, Alonso S, et al. Risk Diagrams Based on Primary Care Electronic Medical Records and Linked Real-Time PCR Data to Monitor Local COVID-19 Outbreaks During the Summer 2020: A Prospective Study Including 7,671,862 People in Catalonia. Front Public Health. 2021;9:693956. Published 2021 Jul 5. doi:10.3389/fpubh.2021.693956

[2] Català M, Pino D, Marchena M, Palacios P, Urdiales T, et al. (2021) Robust estimation of diagnostic rate and real incidence of COVID-19 for European policymakers. PLOS ONE 16(1): e0243701. https://doi.org/10.1371/journal.pone.0243701

[3] Nastos, P.T., Matzarakis, A. The effect of air temperature and human thermal indices on mortality in Athens, Greece. Theor Appl Climatol 108, 591–599 (2012). https://doi.org/10.1007/s00704-011-0555-0

[4] Wang C, Chen R, Kuang X, Duan X, Kan H. Temperature and daily mortality in Suzhou, China: a time series analysis. Sci Total Environ. 2014 Jan 1;466-467:985-90. doi: 10.1016/j.scitotenv.2013.08.011. Epub 2013 Aug 28. PMID: 23994732.

[5] Luo Q, Li S, Guo Y, Han X, Jaakkola JJK. A systematic review and meta-analysis of the association between daily mean temperature and mortality in China. Environ Res. 2019 Jun;173:281-299. doi: 10.1016/j.envres.2019.03.044. Epub 2019 Mar 22. PMID: 30928859.

[6] Royé D, Codesido R, Tobías A, Taracido M. Heat wave intensity and daily mortality in four of the largest cities of Spain. Environ Res. 2020;182:109027. doi:10.1016/j.envres.2019.109027

Supplementary material S2. Formulae used to calculate apparent temperature or heat index

TH(°C)=T(°C)+5*(e-10)/9,

on

$e = RH * e_s / 100$

$$e_s = e_0 \exp\left[\frac{L}{R_v}\left(\frac{1}{T_0} - \frac{1}{T}\right)\right]$$

$e_0 = 0.611$ kPa
$T_0 = 273$ K
$R_v = 461$ J K^{-1} kg^{-1}
$L = 2.5\ 10^6$ J kg^{-1}

Supplementary Table S1. Number of deaths by sex and age groups during the summer months since 2012

	2012	2013	2014	2015	2016	2017	2018	2019	2020	2021	2022
**Total**	**14770 (100%)**	**15018 (100%)**	**15133 (100%)**	**15972 (100%)**	**15558 (100%)**	**14782 (100%)**	**15467 (100%)**	**15066 (100%)**	**15172 (100%)**	**16416 (100%)**	**17551 (100%)**
**0-44 years**	478 (3.2%)	456 (3%)	454 (3%)	418 (2.6%)	393 (2.5%)	362 (2.4%)	384 (2.5%)	346 (2.3%)	341 (2.2%)	347 (2.1%)	329 (1.9%)
**45-79 years**	5651 (38.3%)	5625 (37.5%)	5526 (36.5%)	5696 (35.7%)	5575 (35.8%)	5083 (34.4%)	5438 (35.2%)	5306 (35.2%)	5526 (36.4%)	5747 (35%)	5979 (34.1%)
**>79 years**	8641 (58.5%)	8937 (59.5%)	9153 (60.5%)	9858 (61.7%)	9590 (61.6%)	9337 (63.2%)	9645 (62.4%)	9414 (62.5%)	9305 (61.3%)	10322 (62.9%)	11243 (64.1%)
**Women**	7300 (49.4%)	7441 (49.5%)	7498 (49.5%)	8193 (51.3%)	7672 (49.3%)	7517 (50.9%)	7789 (50.4%)	7537 (50%)	7789 (51.3%)	8136 (49.6%)	8960 (51.1%)
**Men**	7470 (50.6%)	7577 (50.5%)	7635 (50.5%)	7779 (48.7%)	7886 (50.7%)	7265 (49.1%)	7678 (49.6%)	7529 (50%)	7383 (48.7%)	8280 (50.4%)	8590 (48.9%)

Supplementary Figure S1. Z-scores of non-COVID deaths, COVID-19 deaths, ILI cases and temperature by age group and year. MA7 = 7-day moving average

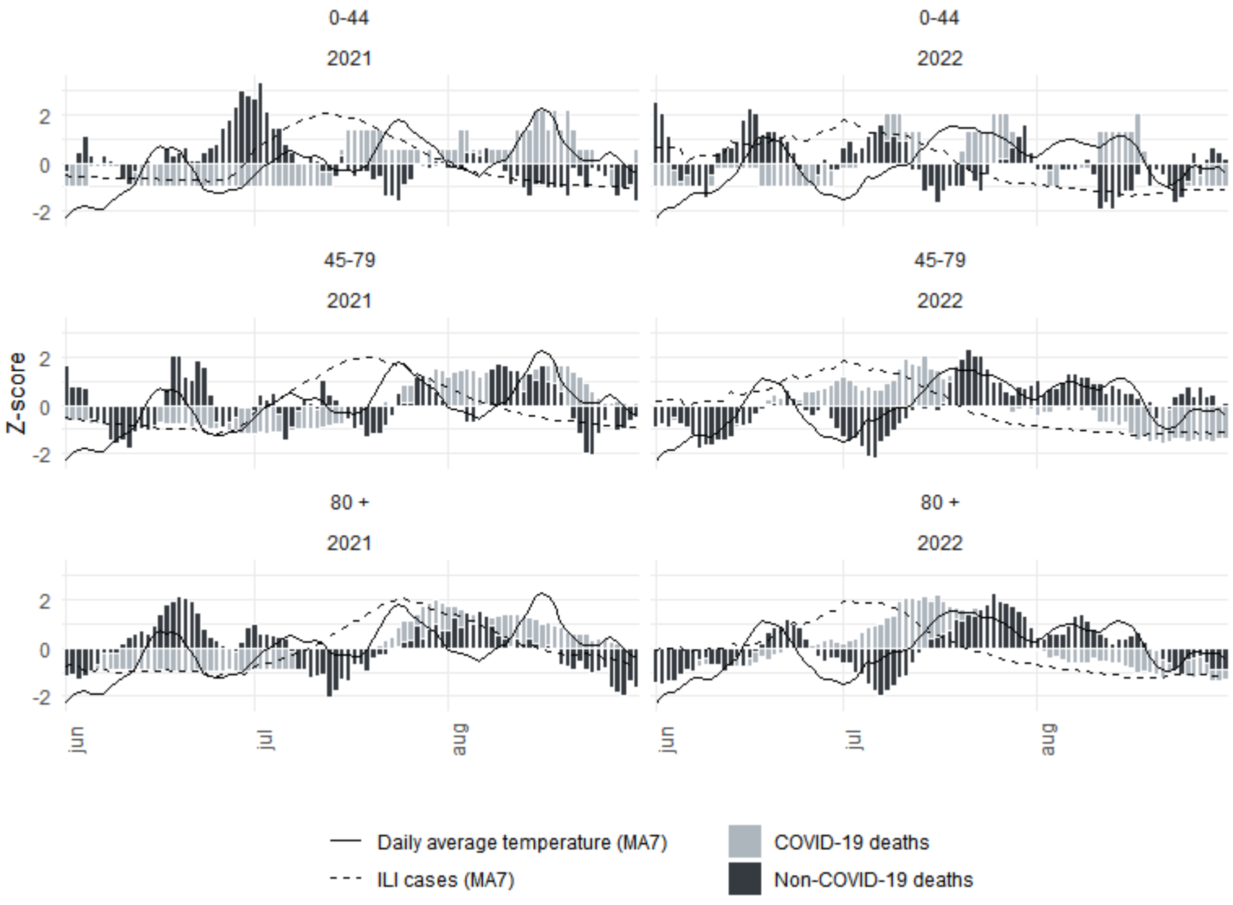

Supplementary Figure S2. Correlations between z-scores of deaths (solid lines) and apparent temperature/ILI cases (dashed lines) by cause of death (all-causes, COVID-19 deaths and non-COVID deaths) during summer 2022, once the theoretical lags have been applied

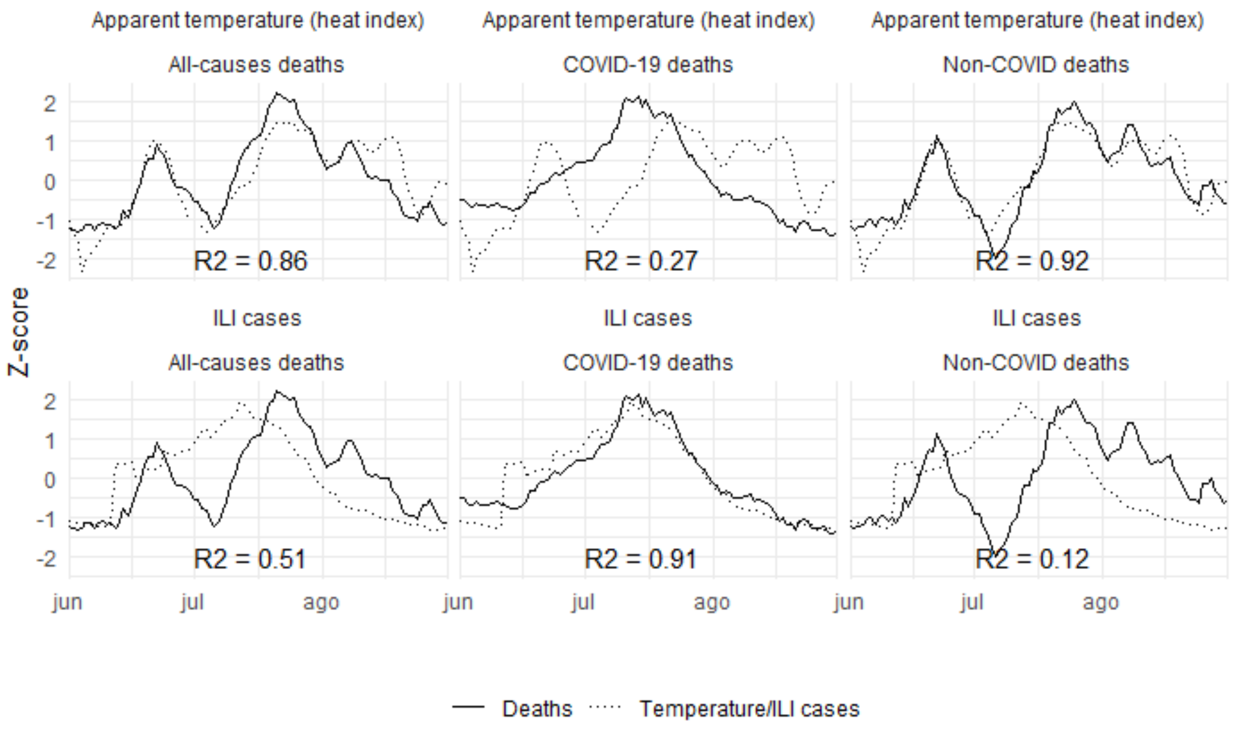

Supplementary Table S2. R2 between the z-scores of ILI cases and deaths and between temperature and deaths (in bold those correlations above 0.8)

Year	Age group	Cause of death	ILI	Temperature
**2021**	**0-44**	All-cause deaths	-0.23	-0.35
		COVID-19 deaths	0.58	0.39
		Non-COVID-19 deaths	-0.35	-0.41
	**45-79**	All-cause deaths	0.62	0.61
		COVID-19 deaths	**0.83**	0.54
		Non-COVID-19 deaths	0.13	0.34
	**>79**	All-cause deaths	0.66	0.47
		COVID-19 deaths	**0.92**	0.50
		Non-COVID-19 deaths	0.07	0.06
	**Total**	All-cause deaths	0.43	0.48
		COVID-19 deaths	0.71	0.51
		Non-COVID-19 deaths	0.07	0.08
**2022**	**0-44**	All-cause deaths	0.20	-0.09
		COVID-19 deaths	0.19	0.43
		Non-COVID-19 deaths	0.15	-0.23
	**45-79**	All-cause deaths	0.02	**0.82**
		COVID-19 deaths	**0.87**	0.20

		Non-COVID-19 deaths	-0.29	**0.80**
	**>79**	All-cause deaths	0.43	0.82
		COVID-19 deaths	**0.91**	0.26
		Non-COVID-19 deaths	0.02	**0.89**
	**Total**	All-cause deaths	0.21	**0.86**
		COVID-19 deaths	**0.86**	0.26
		Non-COVID-19 deaths	-0.19	**0.91**

Supplementary Table S3. R2 between the z-scores of ILI cases and deaths and between temperature and deaths by regions (in bold those correlations above 0.8)

Year	Area	Cause of death	ILI	Temperature
2021	Coastal areas	All-cause deaths	0.47	0.51
		COVID-19 deaths	0.70	0.48
		Non-COVID-19 deaths	0.09	0.06
	Non coastal areas	All-cause deaths	0.28	0.53
		COVID-19 deaths	0.68	0.48
		Non-COVID-19 deaths	0.10	0.46
2022	Coastal areas	All-cause deaths	0.16	0.79
		COVID-19 deaths	**0.86**	0.10
		Non-COVID-19 deaths	-0.28	**0.86**
	Non coastal areas	All-cause deaths	0.47	0.60
		COVID-19 deaths	0.71	0.42
		Non-COVID-19 deaths	0.35	0.61

Supplementary Table S4. Characteristics of the heat waves of 2003 and 2022 (source AEMET[7])

Year	Starts	Ends	Duration	Temperature anomaly[8]	Maximum temperature of the heat wave[9]	Affected provinces
**2003**	2003/06/20	2003/06/23	4	2.2	36.1	17
**2003**	2003/07/30	2003/08/14	16	3.7	37.2	38
**2022**	2022/06/12	2022/06/18	7	3.2	37.7	39
**2022**	2022/07/09	2022/07/26	18	4.5	38.1	44
**2022**	2022/07/30	2022/08/14	16	3.5	36.6	33

[7] Agencia estatal de Meteorología (Aemet). Listado de provincias afectadas por las Olas de calor registradas desde 1975. [Internet]. Available at: https://www.aemet.es/documentos/es/conocermas/recursos_en_linea/publicaciones_y_estudios/estudios/Olas_calor/ListadoProvinciasOlasCalor_1975_2022.pdf [Accessed November 2022]

[8] Average of the maximum temperature anomaly of each station above its temperature threshold (95th percentile of its series of daily maximum temperatures for the months of July and August for the period 1971 to 2000)

[9]Average of the maximum temperatures of the stations having a heat wave for the hottest day

Supplementary Table S5. Characteristics of the excess deaths* of 2003[10] [Simón et al. 2005] and 2022[11] [MoMo, 2022] between 1 June and 31 August in Spain

Year	Age group	Excess deaths (%)
**2003**	All age groups	8%
**2003**	75 - 84 years	15%
**2003**	>84 years	29%
**2022**	All age groups	13.6%
**2022**	75 - 84 years	19.4%
**2022**	>84 years	32.9%

*The excess was calculated as follows: (observed deaths - expected deaths)/expected deaths

[10] Simón F, Lopez-Abente G, Ballester E, Martínez F. Mortality in Spain during the heat waves of summer 2003. Euro Surveill. 2005;10(7):156-161.

[11] The excess mortality in 2022 for each month was 10.3% in June, 32.4% in July and 15.1% in August for the population between 75-84 years and 22.1%, 49.3% and 26.9% respectively for those aged over 84 years.No details about monthly excess mortality are available for 2003
